# A Vexing Question in Motor Control: The Degrees of Freedom Problem

**DOI:** 10.3389/fbioe.2021.783501

**Published:** 2022-01-17

**Authors:** Pietro Morasso

**Affiliations:** Istituto Italiano di Tecnologia, Center for Human Technologies, RBCS Department (Robotics, Brain, and Cognitive Sciences), Genoa, Italy

**Keywords:** redundancy, motor equivalence, embodied cognition, central pattern generators, passive motion paradigm, two/thirds power law, unconstrained manifold concept, equilibrium point hypothesis

## Abstract

The human “marionette” is extremely complex and multi-articulated: anatomical redundancy (in terms of Degrees of Freedom: DoFs), kinematic redundancy (movements can have different trajectories, velocities, and accelerations and yet achieve the same goal, according to the principle of Motor Equivalence), and neurophysiological redundancy (many more muscles than DoFs and multiple motor units for each muscle). Although it is quite obvious that such abundance is not noxious at all because, in contrast, it is instrumental for motor learning, allowing the nervous system to “explore” the space of feasible actions before settling on an elegant and possibly optimal solution, the crucial question then boils down to figure out how the nervous system “chooses/selects/recruits/modulates” task-dependent subsets of countless assemblies of DoFs as functional motor synergies. Despite this daunting conceptual riddle, human purposive behavior in daily life activities is a proof of concept that solutions can be found easily and quickly by the embodied brain of the human cognitive agent. The point of view suggested in this essay is to frame the question above in the old-fashioned but still seminal observation by Marr and Poggio that cognitive agents should be regarded as *Generalized Information Processing Systems* (GIPS) and should be investigated according to three nearly independent but complementary levels of analysis: 1) the *computational leve*l, 2) the *algorithmic level*, and 3) the *implementation level.* In this framework, we attempt to discriminate as well as aggregate the different hypotheses and solutions proposed so far: the optimal control hypothesis, the muscle synergy hypothesis, the equilibrium point hypothesis, or the uncontrolled manifold hypothesis, to mention the most popular ones. The proposed GIPS follows the strategy of factoring out shaping and timing by adopting a force-field based approach (the Passive Motion Paradigm) that is inspired by the Equilibrium Point Hypothesis, extended in such a way to represent covert as well overt actions. In particular, it is shown how this approach can explain spatio-temporal invariances and, at the same time, solve the Degrees of Freedom Problem.

## Introduction

The degrees of freedom problem in motor control states that there are multiple ways for humans or animals to perform a movement to achieve the same goal, leaving the question of how the brain chooses a course of action among infinite ones. The question was explicitly formulated many years ago by (Bernstein, 1967): “It is clear that the basic difficulties for co-ordination consist precisely in the extreme *abundance* of degrees of freedom (DoFs), with which the [nervous] centre is not at first in a position to deal.” Specifically, the human body is characterized by redundancy in many forms: *anatomical redundancy* (in terms of DoFs, muscles and joints), *kinematic redundancy* (movements can have different trajectories, velocities, and accelerations), and *neurophysiological redundancy* (multiple motor units recruited for each muscle); yet such redundancy is not an obstacle to achieve a common goal, according to the principle of Motor Equivalence (Lashley, 1933). In other words, despite such complexity it is quite obvious that the abundance is not noxious at all: in contrast, it is instrumental for motor adaptation and learning, allowing the nervous system the possibility to “explore” the space of feasible actions before settling on an elegant and possibly optimal solution. Ultimately, the crucial question boils down to figure out how the nervous system “chooses/selects/recruits/modulates/stores/recollects” task-dependent subsets of the countless motor variables as functional motor synergies. In any case, human purposive behavior in daily life activities is a proof of concept that solutions can be found easily and quickly by the embodied brain of the “human cognitive agent”, despite this daunting conceptual riddle, A reference point suggested in this essay is to take advantage of the old-fashioned but still seminal observation by (Marr and Poggio, 1976) that cognitive agents should be regarded as *Generalized Information Processing Systems* (GIPS) and should be investigated according to three nearly independent but complementary levels of analysis: 1) the *computational leve*l that is supposed to clarify what needs to be computed and why; 2) the *algorithmic level*, focused on how the computation is organized, in terms of the used representations and the processes employed to build and manipulate the representations; 3) the *implementational/physical level*, related to the selection and activation of the specific neural hardware used to carry out the computation. On the other hand, the view that cognitive agents should be considered as GIPS is in contrast with radically different formulations like the “Smart Vehicles” of (Braitenberg, 1984), the claim of “Intelligence without Representation” by (Brooks, 1991) or the “Radical Embodied Cognitive Science” by (Chemero, 2009). Although intriguing, such a radical approach cannot account, in our opinion, for the large body of knowledge, derived from the field of motor imagery and embodied cognition, supporting the fact that motor cognition cannot be reduced to reactive mechanisms but is a fluid field that holds together real and mental actions in such a way to enable goal-directed actions guided by prospection. In other words, we support the concept that (motor) intelligence is fundamentally dependent on representation.

Thus, we suggest that the age-old degrees of freedom problem should be addressed as a GIPS, employing the three levels mentioned above to discriminate as well as aggregate a number of different hypotheses and solutions investigated in the literature, such as the optimal control hypothesis, the muscle synergy hypothesis, the equilibrium point hypothesis, and the uncontrolled manifold hypothesis, to mention the most popular ones. The analysis also focuses as well on the companion vexing question about the inner structure of *biological motion* revealed by Fitt’s law (Fitt, 1954), the preference of straight trajectories in reaching movements, and the so-called two-thirds power law revealed by general gestures.

This essay focuses initially on the spatio-temporal invariances of multi-joint motor control that stand as a kind of background of the DoF problem and analyzes in some details the main alternative explanations of such invariances developed over the years. Then it focuses on a specific computational model, namely the Passive Motion Paradigm (PMP: Mussa-Ivaldi et al., 1988; Mussa-Ivaldi et al., 1989) that is inspired by the Equilibrium Point Hypothesis (EPH: Feldman, 1966; Feldman, 1986; Bizzi et al., 1992): more specifically, PMP is an extension of the EPH from the “real” elastic force fields, determined by the mechanical properties of skeletal muscles and applied to the “real human body,” to the “virtual” force fields, that express motor intentions/goals/constraints and are applied to an internal model or “body schema.” More generally, this extension implies a view of motor control fully integrated with embodied motor cognition (Mohan and Morasso, 2011; Mohan et al., 2019). The plausibility of this extension is supported by the rather recent consolidation of experimental evidence from motor imagery and the associated revitalization of the ideomotor theory of action, dating back to James’ Principles of Psychology (1890). In particular, it is elucidated how and why the PMP model explains the spatio-temporal invariances and the alternative computational models mentioned above, thus providing a biologically plausible roadmap to solve the DoF Problem. Finally, it is shown how and in which sense the computational framework provided by the PMP model is consistent with the GIPS approach.

## Spatio-Temporal Invariances of Multi-Joint Motor Control

**FIGURE 1:**
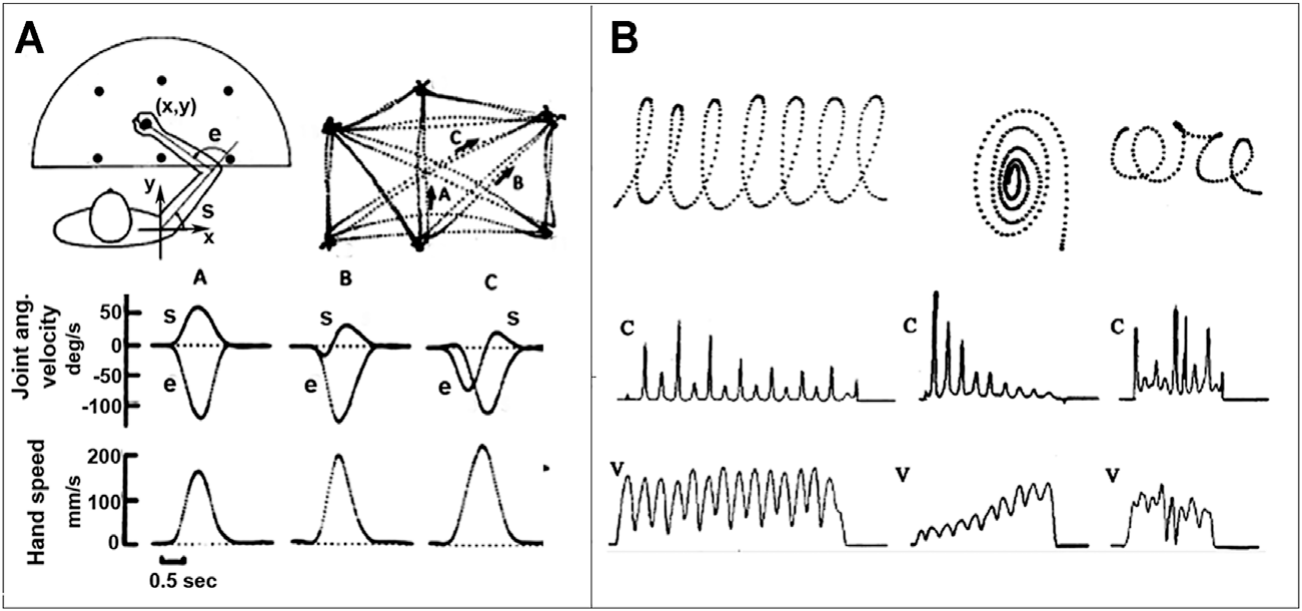
Spatio-temporal invariants in trajectory formation. Panel **(A)**: Planar reaching movements between six target points; A, B, and C correspond to three movement examples, characterized by the joint rotation patterns and the corresponding speed profiles of the joints and the hand; note the invariant bell-shaped speed profiles. Panel **(B)**: Three examples of continuous hand scribbles displayed as digitized trajectories, including the profiles of the velocity (V) and curvature (C); note the anti-correlation of the two profiles.

Until the 70s motor control studies were mostly focused on single-joint control paradigms, within a “reductionist” framework that ignored the “holistic view” implied by the Degrees of Freedom Problem or the Motor Equivalence Principle. The first step towards a more general multi-joint paradigm was the discovery of spatio-temporal invariants, that characterize 2D gestures, such as the following ones (see also Figure 1):

• The bell-shaped speed profile and isochrony of planar reaching movements (Morasso, 1981; Abend et al., 1982). It was found indeed that these movements are approximately straight, with an invariant bell-shape of the hand speed. In contrast, the timing and sync of joint rotation patterns are strongly dependent on the starting position and movement direction. Moreover, for self-paced movements, without specific accuracy requirements, the duration is approximately constant and, thus, peak speed is linearly correlated with target distance.
• The anti-correlation of speed and curvature profiles of movements with multiple *via* points, such as cursive handwriting or drawing gestures (Morasso and Mussa Ivaldi, 1982). When subjects produce continuous hand scribbles, the dynamics and the shape of the movements are not independent, in the sense that the time course of the hand speed and of the scribble curvature are strictly linked: both are characterized by a sequence of peaks and dips that are systematically anticorrelated, in the sense that speed peaks are synchronized with curvature dips and curvature peaks sync with speed dips.

Similar invariants were also found in 3D gestures (Morasso, 1983). In particular, also for 3D hand scribbles the dynamics and the shape of the movements are not independent. The shape of the 3D scribble is characterized by the time course of two parameters: the curvature and the torsion (in 2D scribbles the torsion is null). The peaks of torsion detect when the performing subject changes the virtual plane upon which he/she is producing a given fragment of scribble. The analysis of the movements (Morasso, 1983) showed in particular that the speed/curvature link is preserved and the generated gestures are approximately piecewise planar.

### Alternative Explanations of the Spatio-Temporal Invariances

Discovering that planar hand gestures are characterized by spatio-temporal invariances that mutually constrain shape and kinematics prompted a whole research line, aimed at answering the following questions: Where do such spatio-temporal invariants come from? How can we explain them? How can we simulate their action in a biologically plausible framework? Among the number of different explanations that were proposed we may consider the following ones, listed in chronological order:

• *The 2/3 power law* (Lacquaniti et al., 1983). It addresses the previously mentioned anticorrelation of curvature and speed. In particular, it is shown that in repetitive elliptical scribbles the linkage between the velocity profile v=v(t) and the curvature profile  c=c(t) can be captured by following function^1^: $v\propto \;c^{-1/3}$ . The underlying hypothesis is that such dynamic constraint may be embodied in some specific neural structure that can be recruited, modulating the gain parameter, in order to control the degree of curvedness of the sequence of movements.
• *The minimum jerk model* (Flash and Hogan, 1985). The main point of this theory is that the spatio-temporal invariants of reaching movements can be fully explained by an optimization process that, given the initial and final points of the trajectory and the desired duration, computes the time course of the movement coordinates with the constraint that the hand jerk (the time derivative of the acceleration profile) must achieve a minimum value. The implicit assumption is that the internal neural mechanism that implements this model is a minimization process, operating on the internal representation of the trajectory of the end-effector. The model is limited to 2D motion of the end-effector and does not address the crucial element of the degrees of freedom problem, namely how to distribute the action to the redundant DoFs.
• *The VITE model* (Vector-Integration-To-Endpoint: Bullock and Grossberg, 1988). According to this model, the synergy formation process integrates over time a difference vector (DV), computed from the Target Position and the Present Position of the end-effector, multiplied by a GO-signal that determines the speed profile of the movement aimed to the target. The GO-signal corresponds to a non-linear gating action of the internal representation of the positional error. Also in this case the model is limited to 2D motion.
• *The PMP Model* (Passive Motion Paradigm: Mussa Ivaldi et al., 1988; Mussa Ivaldi et al., 1989). The model provides a force-field-based simulation approach, capable to coordinate implicitly the motion of the end-effector and the corresponding, redundant DoFs. The basic rationale of the model of trajectory formation is the same as the models of motor control based on a force-field approach, namely the idea that multi-joint motor coordination is the consequence of force fields applied to an internal representation of the body, force fields that express goals, intentions, environmental constraints, etc. This idea can be traced back, on one hand, to the EPH (Equilibrium Point Hypothesis: Feldman, 1986; Bizzi et al., 1984; Bizzi et al., 1992) and, on another hand, to the impedance-control schemes proposed in robotics (Hogan, 1985). The whole body is viewed as a network of spring-like elements that individually store elastic potential energy, contributing to a global net potential energy. Considering that energy functions are additive, the global field recapitulates, in a smooth, analogic manner, the complex set of bodily interactions: the result is a “landscape,” with hills and valleys, and thus the overall model will “passively” navigate in the landscape, attracted by the nearest equilibrium configuration, namely a point of minimum potential energy. The minimization of potential energy is a “global” property arising from local interactions, a general concept that has been employed for the design and analysis of large networks (Hopfield, 1982). The PMP applies the concept of “passive motion” to active synergy formation by updating the control input of each element so as to cancel the “stress” induced by a simulated external perturbation, e.g., the attractive force field to a designated target. A recent extension of the PMP model (Mohan and Morasso, 2011) incorporates a gating mechanism, derived from the concept of terminal attractor dynamics (Zak, 1988; Barhen et al., 1989) and analogous to the GO-signal of the VITE model.
• *The Uncontrolled Manifold concept* (Scholz and Schöner, 1999). This approach to solve the degrees of freedom problem generalizes the idea that, for each task, the CNS may select a minimal subset of DoFs that need to be accurately “controlled,” for achieving a given goal, without any specific active control of the remaining DoFs. The idea is that for any task it is possible to subdivide the global configuration space, spanned by the whole set of DoFs, into two orthogonal subspaces: one subspace includes all the joint configurations that lead to the set of values consistent with the successful evolution of the task. This subspace is the Uncontrolled Manifold: motion within this subspace leaves the controlled variables unaffected and thus the control of joint combinations within this manifold is unnecessary. The motion orthogonal to the UCM subspace does affect the controlled variables and thus action planning should only focus on it, with the crucial consequence of reducing the dimensionality of the control problem.
• *Muscle Synergies* (Tresch et al., 1999; Saltiel et al., 2001; D’Avella et al., 2003). The underlying concept of this model of synergy formation is that an efficient solution for addressing the redundancy of the motor control problem might be achieved by representing all useful muscle patterns as combinations of a small number of generators or motion primitives, spanning the muscle activation subspace. This would reduce the dimensionality of the problem and allow sharing neural aggregates across many tasks, allowing the CNS to simplify the control problem by combining discrete elements. Such neural mechanism was investigated first in spinalized or decerebrated animals (Mussa-Ivaldi et al., 1994; Tresch et al., 1999), focusing on the activity of the spinal cord, and then in purposive motor activities of humans (D’Avella et al., 2003), aiming at the detection of correlated patterns of electromyographic activity, specific for each task.
• *Optimal Feedback Control* (Todorov and Jordan, 2002; Scott, 2004; Liu and Todorov, 2007). The Optimal Feedback Control approach (OFC) expands the line of thought initiated by the minimum jerk model. As a matter of fact, OFC is a powerful engineering technique in process control applications, with non-trivial implementation complexity: the theory is fully developed in the case of linear systems, particularly if the cost to be optimized is a quadratic function (of the state and control variables) and a reliable estimate of the state is available. In this case, the optimal control is a linear state feedback law where the control gains are obtained by solving an equation (the Riccati equation), for which robust and efficient algorithms are available. However, if the system to be controlled is characterized by nonlinear dynamics, no unique approach is available and only approximated methods can be devised, to be adapted to the specific task (Beeler et al., 2000). In the application of this design methodology to biological motor control it is necessary to guess the cost function that the brain intends to minimize and implement numerical optimization techniques that are difficult to explain in neural terms. The rationale of the approach is that the best way to engineer a complex control system is to specify a high-level performance criterion and leave the details to “numerical optimization” but while the approach is excellent for accurately fitting the experimental data (Liu and Todorov, 2007) it is of little use to figure out the biological organization of the suggested numerical optimization.
• *The Active Inference perspective* (Friston et al., 2011; Friston and Parr, 2019). This concept rests upon the idea that the brain uses an internal generative model (Jeannerod, 2001) to predict incoming sensory data. Remarkably, this force-based mechanism solves the “Degrees of Freedom problem” in an implicit manner, without explicit kinematic inversion, and it naturally allows to combine multiple goals by superimposing the corresponding force fields. It is worth pointing at the analogy between PMP and Active Inference: in both cases, there is no need to have distinct sensory and motor representations, because the “proprioceptive predictions” of the intended action, generated by the simulation process, are sufficient to allow the motor controller to produce the basic motor synergies. Such predictions encode beliefs about the state of the world, including both proprioceptive and exteroceptive components. The standard causality between sensory and motor representations is somehow inverted: motor commands are not necessarily intended to cause desired movements but desired movements (in the form of the predicted consequences of movement) May cause motor commands.

As better explained in the following, it can be shown that the PMP model explains all the other models listed above and provides a solid computational framework for both human motor neuroscience and humanoid robot cognition (Morasso, 2021). A key point of the paper is that it is impossible to clearly separate motor control from motor cognition. The vast, recent literature on motor imagery allowed to revitalize the traditional Ideomotor Theory, proposed by William James in the 19th century and recently revisited (Shin et al., 2010), namely the concept that the “idea” of an action, i.e., the predicted/desired sensory consequences of covert action, applies as well to goal-directed overt actions and is the internal mechanism that ultimately generates it through the simulation of an internal model (Jeannerod, 2001).

The crucial point is that most theories formulated to account for spatiotemporal invariances in the motor system are “descriptive” of different aspects of the invariances of real actions, by fitting the data with a various degree of accuracy. In this sense, they are all “true” and there is no point in ranking them according to the degree of accuracy or figuring out specific modifications that may increase the accuracy of the predictions. The main goal of the paper, in the GIPS framework, is to outline a plausible approach to characterize a “generative” model that applies equally well to overt and covert actions, in agreement with the simulation theory of action formulated by Marc Jeannerod (2001). As shown in the rest of the paper, PMP appears to match this requirement in a simple “economic” manner, providing a uniform computational mechanism for both covert and overt actions that is complex but not too complex. The OFC and the muscle synergy models may also be considered “generative” but fail the requirements above in two crucial aspects: 1) they are unable to apply to overt and covert actions in a uniform way; 2) computationally, OFC is too complex to be biologically plausible and the muscle synergies model is too simple, because is a kind of table-lookup mechanism, based on a linear combination of pre-recorded high-dimensional patterns).

Before proceeding in the analysis of the PMP model, it is worth to clarify what is the specific meaning of the word Synergy in the context of this paper. As a matter of fact, the DoF Problem and the Synergy Concept are the two faces of the same coin: the human body has too many DoFs and too many muscles to allow the brain to control all of them independently. In any case, the real function of the brain is not the control of movements per se but the organization of purposive actions, identified by a small number of control variables (thus reducing dimensionality) and structured according to the principle of compositionality: this means that humans simplify the generation of various motor behaviors through the re-use of a limited number of basic motor primitives to be combined in an additive manner, rather than developing entirely new modules for each behavior/task. If we consider the etymology of “synergy” (the word derives from two ancient Greek words: συν+εργός, Sun + ergòs, i.e., “working together”) it is not surprising that anybody working in motor neuroscience agrees on its fundamental role in the organization of purposive actions, although it is equally evident that in the literature there is a large variety of synergies (or “zoo of synergies” to quote Mark Latash, 2008): kinematic synergies (Freitas et al., 2006; Huang et al., 2021), kinetic synergies (Slomka et al., 2015), muscle synergies (Cheung and Seki 2021), to name a few. They all clarify the concept that the DoFs are not independent but are recruited by combining a limited number of adaptable primitives. The muscle-less synergies advocated in this manuscript may also be considered “ideomotor synergies” and their rationale is based on the equivalence between overt actions (that imply the generation of muscle patterns) and covert actions (that are muscleless by definition). The working hypothesis that muscleless synergies are “primitives on the top of the computational chain” does not contradict the evidence that low-level coordinative structures, possibly encoded by spinal premotor interneurons, exist and are recruited during overt actions. The frequently invoked need of a verified neural basis of muscle synergies is descriptive of the correlation among different neural processes but does not imply a causal relationship: in our opinion, it is not a plausible computational process capable to generate the observed correlations in overt actions and, at the same time, available to the brain for prospection in covert actions. The underlying issue is that, in an embodied cognitive framework, motor cognition and motor control of purposive actions are indeed different neural processes but they must share a common representation of action. A further point to be clarified is related to the specific meaning of “motor primitives” and the nature of the compositional process that allows them to be combined. This point will be clarified in the section related to GIPS.

## How the PMP Model Explains Spatio-Temporal Invariances

Let us consider the basic form of the PMP model, which is focused on planar reaching movements but promptly generalizes to 3D movements, from a starting point $P_{0}$ to a target point $P_{T}$ . The movements are driven by an attractive, virtual force field F , centered in the target position $P_{T}$ and applied to the moving end-effector p(t) :

$$F\left(t\right)=K\left(P_{T}-p\left(t\right)\right)$$ (1)

**FIGURE 2:**
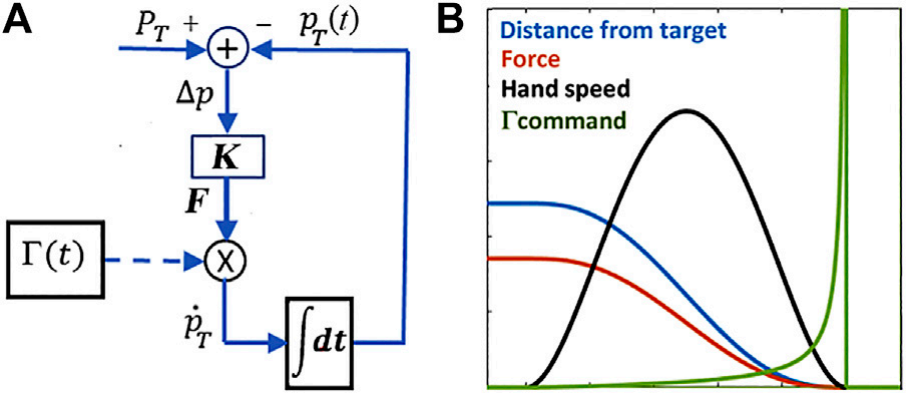
**(A)**: PMP model for the generation of a $p_{T}\left(t\right)$ moving target aimed to the final target $P_{T}$ . The virtual force field F is gated by the *Γ*-function that indirectly control the speed profile ${\dot{p}}_{T}\left(t\right)$ . **(B)** shows that the spiky Γ-command induces a bell-shaped speed profile with the corresponding smooth reduction of the distance from the target.

If the matrix K is proportional to the unitary matrix, the force field is isotropic and, by applying it to the end-effector, the hand will follow a straight path terminating in the target point. However, since the field intensity vanishes as the end-effector approaches the target, the time to target is virtually infinite and the velocity profile is far from bell-shaped. An indirect control of the timing, used by the PMP model, is obtained by a non-linear gating mechanism, namely the *Γ*-function of Figure 2: this function is null before the initial time instant $t_{0}$ and grows smoothly but very quickly until the designated final time $t_{f}=t_{0}+T$ , where it diverges to infinity before collapsing to 0. The purpose of the Γ-function is to set a hard deadline to the gradient descent process seeking an equilibrium state, after the initial disequilibrium induced by the instantiation of a target, whatever the dimensionality of the underlying system and the distance of the target from the initial state. Among the different forms that can be used for this gating mechanism, the one adopted by the PMP model is defined as follows:

$$\{\begin{array}{c} \Gamma =\frac{\dot{\text{ξ}}}{1-\text{ξ}}\;\;\;\mathrm{for}\;0<t-t_{0}<T\;\;\\ \Gamma =0\;\;\;\;\;\mathrm{for}\;\;t-t_{0}\leq 0\;\;\mathrm{and}\;\;t-t_{0}\geq T \end{array}$$ (2)

where $\text{ξ}=6\;\gamma^{5}-15\;\gamma^{4}+10\;\gamma^{3}$ is a smooth 0→1 transition and γ is the normalized time: $\gamma =\frac{t-t_{0}}{T}$ .

The *Γ*-function does not impose the speed profile but forces the gradient descent driven by the force field to achieve equilibrium in finite time. Figure 2 shows an example of reaching movement generated by Eq. 1 and Eq. 2, according to the block diagram in the left panel of the figure; the right panel shows the time course of the Γ-function and demonstrates that the PMP model can indeed induce a smooth acquisition of the target (the distance monotonically decreases to zero) with a symmetric bell-shaped speed profile, without the explicit optimization suggested by the minimum jerk model. Moreover, the PMP model described above is not limited to straight trajectories: curved trajectories can be generated by the same block diagram of Figure 2 if the gain matrix K includes a rotational component, without affecting the terminal attractor properties of the model. The model can be used iteratively in such a way to generate a sequence of PTP (Point to Point) movements: the final point of each movement becomes the initial point of the next one, provided that the Γ-functions of consecutive commands are not overlapped in time, namely the initial instant of each command occurs later than the final instant of the previous one.

**FIGURE 3:**
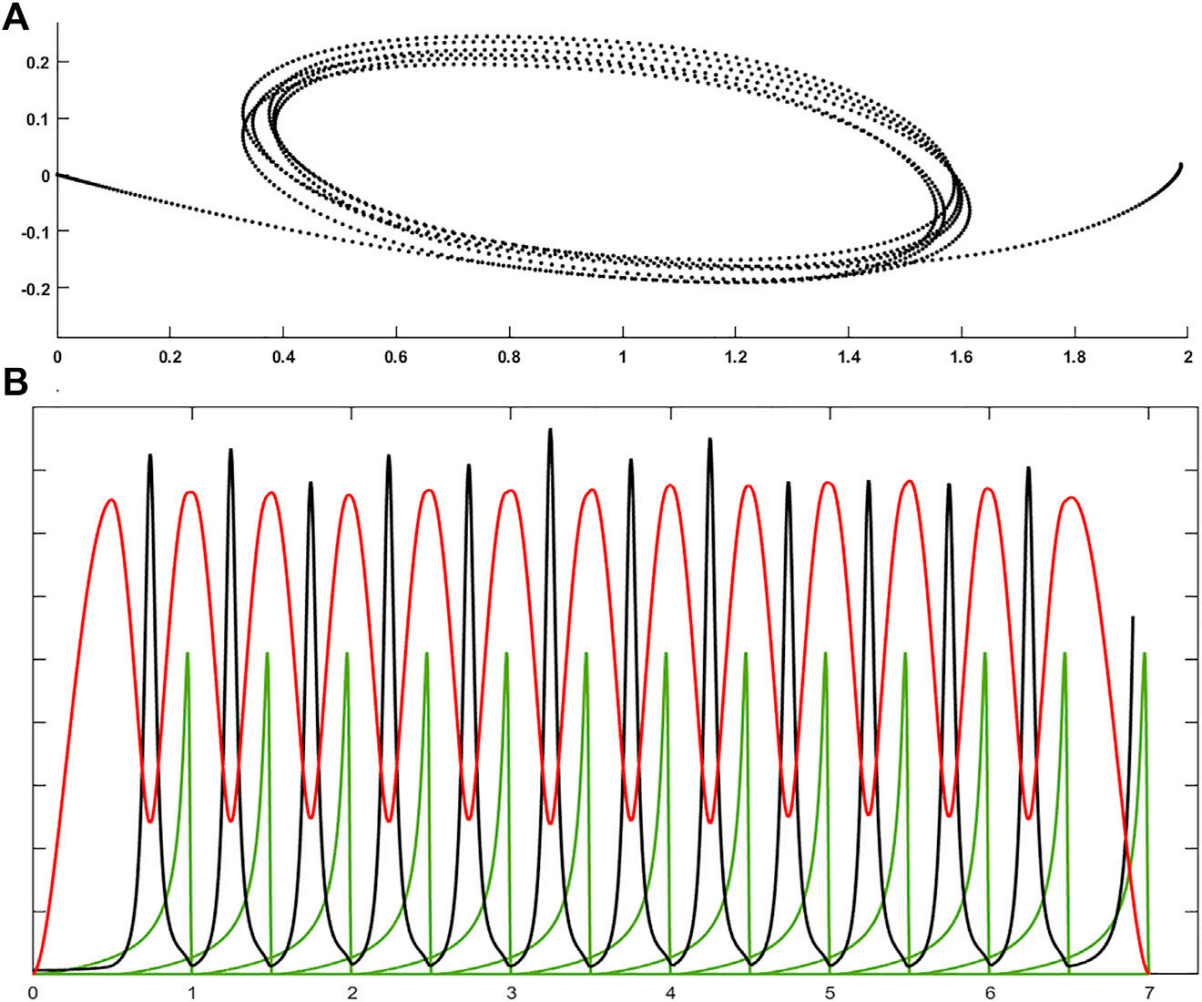
**(A)**: Example of a generic trajectory with multiple VPs generated by the PMP model by chaining a sequence of PTP movements with time overlap between consecutive *Γ*-functions; the trajectory is characterized by 13 targets and 12 VPs; the *Γ*-functions of successive commands have a 50% time overlap. **(B)**: time course of speed (red), curvature (black) profiles, and the 13 *Γ*-functions (green). Each *Γ*-function has a duration of 1 s, with a 50% overlap between successive functions.

The model can also be extended to any VP trajectory, i.e., trajectory with multiple *Via* Points, as in cursive handwriting or hand drawing, in a very simple and natural way. The reason is that the PMP model is based on elastic force fields and we should consider that the corresponding energy functions are additive. Thus, a generic trajectory with multiple VPs ca be generated by chaining a sequence of PTP movements with time overlap between consecutive *Γ*-functions. Figure 3 shows an example, characterized by 13 targets and 12 VPs: the targets are alternated back and forth on the horizontal axis, with approximately equal distance (a small random displacement is added for improving the graphical rendering); the force field of each PTP movements is equally curved; the *Γ*-functions of successive commands have a 50% time overlap. The result is a sequence of elliptical shapes, except for the first and the last curved movement (top panel of Figure 3), and the remarkable feature of the generated trajectory with multiple VPs is clarified by the bottom panel of Figure 3, that plots the speed and curvature profiles, together with the sequence of Γ-functions: speed and curvature are clearly anti-correlated. The VPs are the points of peak curvature but these points are not explicitly expressed: they are implicitly generated by the systematic overlapping strategy of the chaining procedure. Moreover, by plotting speed vs. curvature in a logarithmic scale it is possible to demonstrate that the PMP model applied to a sequence of overlapped VPs matches the 2/3 power law mentioned above without an explicit implementation of the law in a neural controller of the synergy formation mechanism. Rather, the correlation of shape and kinematics implied by the law is simply the computational consequence of the repetitive application of the simple PMP mechanism with an overlap between two consecutive motor commands. In summary, the simulation of the basic form of the PMP model is capable to reproduce, at the same time, the minimum jerk hypothesis, without any optimization mechanism, and the 2/3 power law, without any explicit figural-timing constraint.

## How the PMP Model Solves the Degrees of Freedom Problem

In the previous section, it was shown how the PMP model can reproduce the spatio-temporal invariances of multi-joint motor control, focusing on the kinematics of the end-effector. This formulation neglected how the described neural model might be integrated with the recruitment of the redundant DoFs of the human body, namely the key point of the degrees of freedom point. Mapping the planned trajectories of the end-effector onto the redundant, articulated joint network is usually called inverse kinematics, a typically ill-posed transformation due to the kinematic redundancy of the human body. This means, in particular, that the inverse transformation can have infinite solutions or no solution at all. The rationale of the PMP approach is to avoid this critical problem by focusing on force rather than on motion, thus dealing only with a network of well-posed transformations.

For example, if we wish to induce a small displacement of the end-effector $\Delta p_{\mathrm{ee}}$ from a given equilibrium point and attempt computing the incremental joint rotation Δq that allows the desired displacement to occur, we run into the trouble of inverse kinematics, i.e., an ill-posed transformation. However, it is possible to avoid such problem by using the force field-based approach described in the previous section: instead of forcing the system to carry out the desired incremental motion $\Delta p_{\mathrm{ee}}$ , we may “disturb” the current equilibrium with a force field attracting the end-effector in the same direction: $\Delta F_{\mathrm{ee}}=K_{\mathrm{ee}}\;\Delta p_{\mathrm{ee}}$ . This disturbance $\Delta F_{\mathrm{ee}}$ can always be mapped from the end-effector space to all the DoFs of the joint space, giving a unique solution $\Delta \tau =J^{T}\Delta F_{\mathrm{ee}}$ , where J is the Jacobian matrix of the non-linear, redundant kinematic transformation p=f(q) . In other words, while the transformation in terms of incremental motion $\left(\Delta p_{\mathrm{ee}}\to \Delta q\right)$ is generally ill-posed, the corresponding transformation in terms of forces $\left(\Delta F_{\mathrm{ee}}\to \Delta \tau \right)$ is well-posed and admits a unique solution as a consequence of the principle of virtual works.

The crucial step, at the heart of the PMP approach, is then to apply the concept of “passive motion”, that consists in updating the state of each joint so as to cancel the “stress” induced by the simulated external perturbation: Δq=C Δτ , where C is a square matrix that distributes the “passive motion” induced by the virtual disturbance to all the joints. This incremental motion in the joint space can then be mapped uniquely to the end-effector space, using again the Jacobian matrix: Δp^ee=J Δq . Summing up, the PMP model avoids the ill-posed inverse kinematic transformation $q=f^{-1}\left(p\right)$ by the following chain of transformations that are all well-posed from the end-effector space to the joint space and then back to the end-effector space:

$$\Delta p_{\mathrm{ee}}\to \Delta F_{\mathrm{ee}}\to \Delta \tau \to \Delta q\to \Delta {\hat{p}}_{\mathrm{ee}}$$ (3)

**FIGURE 4:**
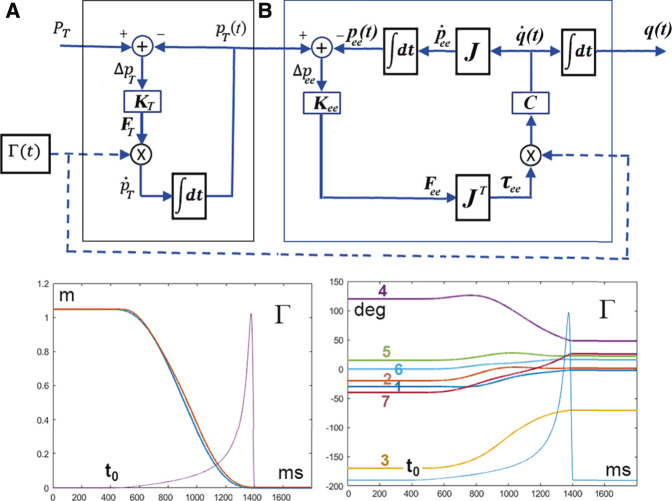
**(A)**: Extended PMP model for solving the Degree of Freedom problem; the module A generates the moving target ${\dot{p}}_{T}\left(t\right)$ **,** attracted to the final target point by the force field $F_{T}$ ; the module **(B)** generates the trajectory of the end-effector $\left(p_{\mathrm{ee}}\left(t\right)\right)$ and distributes the action to all the DoFs (q(t)) by using the force field $F_{\mathrm{ee}}$ . In the lower part of the figure the left graph shows the time course of the distance from the final target point of the moving target (red curve) and of the end-effector (blue curve); the right graph shows the time course of the 7 DoFs (yaw-pitch-roll of the wrist, pitch of the elbow, and yaw-pitch-roll of the wrist); both graphs also include the common Γ-command.

Ideally, $\Delta {\hat{p}}_{\mathrm{ee}}$ should be equal to $\Delta p_{\mathrm{ee}}$ but this is not the case in general because the stiffness matrix of the end-effector is anisotropic: the natural solution is to close the loop of the chain of transformations indicated above by redirecting the force field to the designated target at each time instant, as shown in Figure 4 (top panel). The figure clarifies the fact that the Passive Motion Paradigm is split into two modules: a module (A) that generates a moving target $p_{T}\left(t\right)$ , terminating in the final target $P_{T}$ , and a module (B) that moves all the DoFs in such a way to keep the end-effector $p_{ee}\left(t\right)$ as close as possible to the moving target. Both modules are driven by force-fields and thus in the PMP model there is a concurrent “double pulling force”: from the moving target to the final target $\left(F_{T}\right)$ and from the moving target to the moving end-effector $\left(F_{ee}\right)$ . This aspect of the PMP model is supported by experimental evidence linking the maintenance of posture in a multijoint system to that of generating a movement: it was found indeed that the CNS does not apply a final position control mechanism but programs a reaching movement by shifting the equilibrium position of the hand toward the target in a continuous manner (Bizzi et al., 1984; Shadmehr et al., 1993). In the PMP model, the action of the two force fields is synchronized by gating both of them with the same *Γ*-function: the bottom panel of Figure 4 shows that both the moving target point $p_{T}\left(t\right)$ and the end-effector $p_{ee}\left(t\right)$ reach the final target point $P_{T}$ at the same time, dictated by the *Γ*-function. Moreover, the figure illustrates how the PMP model solves the DoF problem, distributing smoothly the action among all the DoFs in an implicit way.

The biological plausibility of the model described above is also supported by experiments on the coordination mechanisms underlying bimanual reaching. Apart from the spatio-temporal invariance of multi-joint reaching movements, exemplified by the bell-shaped velocity profile, independent of starting posture, movement direction and target distance, we should also consider the speed-accuracy trade-off, elucidated by the Fitt’s law (Fitts, 1954): the duration of reaching movement grows with the required index of difficulty, namely the ratio between target distance and target size. In the PMP model this means that the CNS should choose the duration of the *Γ*-function in accordance with the expected difficulty index. But what about bimanual reaching? Unless the two targets have the same difficulty, the Fitt’s law would predict different reaching times. However, this is not what happens: the experiments by (Kelso et al., 1979) clearly show that bimanual motions reach the targets simultaneously and the common duration is dictated by the motion with the higher difficulty index. The picture that emerges from such studies of interlimb coordination is that the role of central control patterns is not to prescribe the full details of the intended action but rather to organize functional groups of DoFs, also known as coordinative structures (Turvey, 1977) or motor synergies. An extension of the PMP model for covering bimanual coordination is quite straightforward: it is sufficient to instantiate two copies of the model of Figure 4 for the two arms (A_left_, B_left_, A_right_, B_right_) and synchronize the four modules with the same *Γ*-command. Other studies investigated bimanual coordination, for example manipulating a nonholonomic car (Tsuji et al., 2002) and it was found that the timing of the coordinated movements is compatible with a non-linear gating model based on a TBG (Time Based Generator) quite similar to the *Γ*-command.

As regards the motor synergies investigated by (D’Avella et al., 2003), they clearly fit the quest, initiated by Bernstein, for computational mechanisms capable to reduce the complexity of motor control. However, demonstrating the low dimensionality of patterns of electromyographic activities embedded in actions of daily life is not sufficient to conclude that the small number of high-dimensional muscle activation patterns “are” the sought “motor primitives” to be combined for synergy formation in general. This hypothesis is in contrast with the large body of research on motor imagery that supports the fundamental equivalence of mental and real motor actions, including the timing aspects: Decety and Jeannerod (1995) showed that imagined discrete movements obey Fitts’s law and their durations are well correlated with those of actual movements; Karklinsky and Flash (2015) demonstrated that imagination of continuous scribbles is consistent with the two-thirds power law of real scribbles. Thus, in our opinion a more appropriate approach to synergy formation is not based on “muscle synergies” but on “muscle-less synergies” or “ideomotor synergies” (Mohan et al., 2019) represented and generated by the PMP model. In this framework, the muscle synergies are not the motor primitives on the top of the computational chain but the results of the force-field driven internal simulation carried out by the PMP model. The motor primitives are thus the recruited force fields and the muscle synergies, evoked only in overt actions, are the visible consequences: the dynamic effects of the interaction between a muscle-less mental synergy and the control modules recruited for a specific task, as a combination of feedforward and feedback control mechanisms, in conjunction with coactivation patterns of muscle activity for modulation of joint stiffness.

For the UCM concept, we may observe that it is incorporated intrinsically in two crucial elements of the PMP model illustrated in Figure 4. First of all, the mapping of the virtual disturbance from the end-effector space (i.e., the task space) to the joint space (or DoF space) is indirectly and intrinsically ranking the whole set of DoFs according to the relevance of each DoF for the planned action: $\Delta \tau =J^{T}\Delta F$ **.** The rank of each DoF dynamically changes during the movement and we may estimate the relative weight of each DoF $\left(w_{i}\left(t\right)\right)$ , at each time instant, with the following indicator: $w_{i}\left(t\right)=\Delta \tau_{i}\left(t\right)/\left|\Delta \tau \right|$ . In particular, the DoF with the highest ranking will be the one that yields the most to the virtual disturbance. The second elements of the PMP model that may be associated to the UCM concept is the compliance matrix ***C*** that maps the disturbing torque absorbed by each DoF into the corresponding incremental displacement: Δq=C Δτ **.** This may increase or decrease the ranking of each DoF and thus influence the clustering of the whole set of DoFs in the controlled and uncontrolled manifolds. Therefore, in the PMP framework there is no need to explicitly specify the two alternative manifolds: the border between the two clusters is rather fuzzy and time varying, due to the complexity of the body kinematics and the task-dependent requirements.

A further issue that is related to the UCM concept, on one side, and to the additivity of force fields acting on complex kinematic networks, on another side, is the possibility to integrate in the dynamics of PMP models additional force fields that may express additional constraints or task requirements. An example is the “regularization” of the synergy formation process aimed at satisfying the limited range of motion of each joint. This is a constraint that would be very difficult to formulate in an exact treatment of the inverse kinematic problem. In the PMP framework it is sufficient to introduce an additional force field, in the joint space, with a non-linear repulsive action from the joint limits of each DoF. This force field may be added to the attractive force field, defined in the end-effector space for expressing the target reaching intention, and possibly to other force fields for expressing additional constraints or requirements. It is important to note that all such force fields may be defined in different spaces with different reference frames and thus the PMP model can be designated as a *multi-referential system of synergy formation*. However, the effects of the different force fields converge ultimately to the joint reference system, coordinating the motion of the whole set of DoFs, using a complex network of well-posed transformations. The crucial point of synchronizing the effects of all the different components is implemented, as shown in Figure 4, by gating the different perturbing sources with the same *Γ*-function. This also clarifies the fact that the abundance of DoFs is functionally essential for achieving at the same time the multiple sub-goals of a given task: for example, reaching a target at a given time, while keeping each joint inside its range of motion and avoiding to hit dangerous obstacles in the environment. A possible neural implementation of the Γ-function is suggested by studies that document the gating action of the basal ganglia on the activation of the motor cortex. For example (Horak and Anderson, 1984), found that two nuclei of the basal ganglia (the Substantia Nigra pars reticulata and part of the Globus Pallidus) carry out a gating and velocity scaling action of the commands sent to the motor thalamus and precentral cortex.

In summary, the PMP model integrates in the same computational framework the spatio-temporal invariants, described by the minimum-jerk model, VITE model, and two-thirds power law, as well as the coordination requirements of redundant DoFs, expressed by the UCM model and the muscle synergies.

## The Generalized Information Processing Systems Approach

### The Computational Level

A computational theory for addressing the Degrees of Freedom Problem should stem from the fact that a cognitive agent is continually involved in prospectively guided, goal-directed actions that involve the whole body and thus is faced by the challenge of choosing an action course that recruits the degrees of freedom on the basis of the desirable and predictable outcome. Thus, the core of the theory, namely the definition of “what needs to be computed and why,” is a mechanism that allows the brain to integrate in the same process the capability to shape the motor system in anticipation to execution as well as the awareness of the feasibility of potential actions without execution. Moreover, this internal model should incorporate a knowledge about the causal relationship between the task spaces (related to the designated “end-effectors”) and the joint space (the DoF space): more specifically, it should be able to predict the incremental displacements in the task space determined by arbitrary variations in the joint space as well to compute the joint efforts capable to equilibrate virtual perturbations applied to one of the end-effectors of the body in the task space. Mathematically, such computations are equivalent to the Jacobian matrices that link the joint space and the task spaces. The computation of such Jacobian matrices is a key element for solving the DoF problem because they allow to rank in a direct and implicit way all the DoFs of the body for a given task, expressed as a set of virtual force fields applied to the end-effector.

Another requirement of the computational theory is that the theory must not be purely descriptive but provide robust generative capabilities with cognitively penetrable features: this means that the details of the simulation process that generates ideomotor synergies should be relevant to cognitive processes related to prospection, learning and decision making.

The other key element of the theory is that it must capture the spatio-temporal invariants that characterize human actions, independent of the number of involved end-effectors and DoFs. The crucial function of the invariants is to allow the process of coordination of the redundant DoFs to evolve in a smooth and coherent manner, by relying on the composition of complex gestures in terms of simpler sub-actions or motion primitives. The solution to this is to integrate in the network of Jacobian matrices, that represents the internal model of the whole body, a non-linear gating mechanism applied to the virtual force fields in such a way to dynamically synchronize all the elements of the network, in analogy with the recall of information in large associative networks, without any need of a universal clock or metronome.

The theory covers the motor cognitive aspects of synergy formation with a mechanism of recruitment of the redundant DoFs and of synchronization of motor primitives that allows smooth composition. Thus, the theory fully represents the organization of covert or mental actions but it does not and must not cover specific control aspects in action execution that are determined by the physical interaction of the body with the environment: action execution, in addition to a well-structured ideomotor synergy, will also require a combination of different control mechanisms (feedforward, feedback, and stiffness control) which are outside the scope of the computational theory.

The PMP model is intended to answer the above requirements of the computational level formulation of the DoF problem, although alternative formulations cannot be excluded. However, the different models analyzed before for explaining the spatio-temporal invariants do not fit the computational requirements except for the Active Inference model, as previously observed, and the VITE model but only for the non-linear gating mechanism. The minimum jerk model and the 2/3 power law are more descriptive than generative and, in any case, do not address the redundancy issue of the DoF problem. The UCM model is mainly descriptive, leaving open the question of dynamically sorting the set of DoFs in relevant and non-relevant subsets for a given task. The muscle synergy model, as well as the OFC model, only apply to the control part of overt actions and, in any case, they are far from being cognitively penetrable. For a computational theory of this kind, the classical planning-acting-sensing loop is not appropriate because it is impossible to separate logically in a clear manner the three components and sequence them in time.

### The Algorithmic Level

In agreement with the computational theory defined above, we suggest that the algorithmic level of analysis of the DoF problem should be based on the simulation theories (Jeannerod, 2001; O’Shea and Moran, 2017) and emulation theories (Grush, 2004; Ptak et al., 2017) for the representation of prospectively guided, goal-directed actions. This point of view is strongly supported by following statement by (Marc Jeannerod, 2001): “The possibility to experimentally access to cognitive or mental states characterized by absence of overt behavior represents a new avenue for neuroscience.” From this derived the hypothesis that the motor system is part of a simulation network that is activated under a variety of conditions in relation to action, either self-intended or observed from other individuals. This is indeed the starting point for the introduction of the PMP model which addresses the DoF problem by factoring it out in two main components of synergy formation: 1) giving shape to the synergy, by superimposing multiple virtual force-fields, and 2) giving rhythm to the synergy with a suitable gating and velocity modulation. The PMP model relies on a network of Jacobian matrices that correspond to the different kinematic chains of the human body, including a mechanism of serial/parallel connections. The basic algorithmic function is the simulation of the network. The algorithmic level of analysis for addressing the DoF problem is also appropriate for clarifying the concept of motor primitive, as the basic cellular element to be combined in order to generate general actions. In contrast with the common wisdom, typical for example of popular methods of movement notation, such as Labanotation (Laban, 1956), Therblig notation (Aft, 2000) or the Human Action Language (Guerra-Filho and Aloimonos, 2007), that identify motor primitives with elementary movements, we think that it is more appropriate to assume that motor primitives are force fields. The basic (algorithmic reason) for this assumption is that in a complex mechanical network, like the human body, force fields are additive while elementary movements are not.

### The Implementation/Physical Level

The algorithmic hypothesis, that the same internal model is involved in the generation of overt and covert actions, can lead to different implementation strategies for transforming a selected covert action into the corresponding overt counterpart. The underlying hardware that is supposed to allow the neurobiological implementation of an internal mechanism similar to the PMP model must count onto two main modules: 1) a module for representing the Jacobian matrices and 2) a module for the control of timing and synchronization.

A biomimetic approach for defining and implementing the former module is the process of *sensorimotor babbling*. This is an idea that was originally proposed by (Piaget, 1952) for the study of sensorimotor development in children. He described a “primary circular-reaction hypothesis” supported by the fact that newborn infants repeatedly perform exploratory movements which are “centered on themselves” rather than driven by external stimuli. Such empirical observations prompted lines of research, both in developmental psychology (von Hofsten, 1982) and computational neural modeling (Kuperstein, 1988), that viewed “motor babbling” inherent to primary circular-reactions (e.g., the performance of random hand movements in front of the eyes) as a crucial mechanism for enhancing the formation of associations between efferent motor patterns and re-afferent perceptive/proprioceptive patterns. More recently, motor babbling was applied to robotics in order to promote learning the internal representation of the body: Hersch et al. (2008) proposed an algorithm enabling an embodied robot to visually learn its body schema, by visually observing its end-effectors when moving them; Sturm et al. (2009) developed a model based on Bayesian networks that allows a robot to simultaneously identify its kinematic structure and learn the geometrical relationships between its body parts as a function of the joint angles. Moreover, the babbling-based approach was also extended for the internal representation of the use of tools (Bhat et al., 2017), considering the underlying neurophysiology about the adaptation of the receptive fields of skilled users (Maravita and Iriki, 2004).

As regards the module for the control of timing and synchronization of multiple sensorimotor processes, the previously defined *Γ*-function or GO-signals are specific examples. A more detailed analysis of the *Γ*-function is provided by the TBG (Time base Generator) model (Tsuji et al., 2002) that allows a parametrization of the function, in order to describe small modifications of the symmetry of the bell-shaped velocity profile that are consistently associated with any individual (Kittaka et al., 2020). This method of analysis is also applicable in the clinical field by using it for the quantitative evaluation of the Trail Making test (Sakai et al., 2021): this is a neuropsychological test which is widely used to assess the cognitive function of patients with motor-cognitive impairments, as in the case of stroke.

In general, we suggest that the *Γ*-function may be considered as a member of the large family of CPGs (Central Pattern Generators), although this kind of neural mechanisms have been investigated mainly for explaining the neural drive of rhythmic and stereotyped motor behaviors like walking, swimming, breathing, chewing, swallowing. Although it is generally assumed that CPGs, typically located in the spinal cord and brain stem, are characterized by the ability to operate with a minimal intervention of higher brain areas, still they require modulatory inputs in order to perform in a flexible way. The role of CPGs in less stereotyped motor behaviors, characterized by a clear cognitive interaction, has been clarified in the field of speech motor control (Barlow et al., 2010; Rusaw, 2013) or sign language (Tkachman et al., 2021). Reaching movements seem to be far away from the area of motor control related to rhythmicity related to CPGs. However, cursive handwriting, scribbles or hand gestures in dance strongly support the view that the observed motor patterns may be the result of the superposition of motor primitives similar to PTP movements, with a clear rhythmical and prosodic structure that may imply a pattern generator, in charge of timing. Such CPG clearly cannot be localized downstream the neuroaxis as in the case of locomotion and there is diffused evidence, summarized by (Bullock et al., 1999), that it may involve thalamo-cortical loops with the purpose of gating and velocity scaling. In any case, we suggest that the widespread use of CPGs for coordinating more or less rhythmic behaviors of the highly redundant motor system is one of the main techniques adopted by the CNS to tame the degrees of freedom problem. This consideration is also consistent with the evolutionary analysis of CPGs reported by (Katz, 2016).

## Conclusion

In conclusion, we wish to emphasize our complete agreement with the view by (Latash, 2012) that there is no “problem” of motor redundancy; rather there is the bliss of motor abundance that allows humans as well as members of other species to exhibit adaptive behaviors across a variety of conditions, in a changing and challenging environment. This is at the heart of what (Latash, 2012) denotes as “good variance” of human behavior, in contrast with the “invariance” (and consequent inflexibility) of exact algorithmic models. We only observe that such good variance and the bliss of motor abundance are made possible by the strategy of factoring out shaping and timing, described in this essay.

## Data Availability

The raw data supporting the conclusion of this article will be made available by the author, without undue reservation.

## Footnotes

1 An equivalent formulation of this function is as follows: $\omega \;\propto \;c^{2/3}$ , where ω is the instantaneous angular velocity of the hand. This clarifies where the “2/3” element comes from.
